# Regenerative Medicine for Knee Osteoarthritis – The Efficacy and Safety of Intra-Articular Platelet-Rich Plasma and Mesenchymal Stem Cells Injections: A Literature Review

**DOI:** 10.7759/cureus.10575

**Published:** 2020-09-21

**Authors:** Hoi Leng Ip, Debashis Kumar Nath, Safia H Sawleh, Md. Humayun Kabir, Nusrat Jahan

**Affiliations:** 1 Internal Medicine, California Institute of Behavioral Neurosciences & Psychology, Fairfield, USA

**Keywords:** knee osteoarthritis, platelet-rich plasma, autologous conditioned plasma, mesenchymal stem cells, bone marrow, adipose tissue, umbilical cord, intra articular injection, regenerative medicine, western ontario and mcmaster universities arthritis index (womac)

## Abstract

The prevalence of osteoarthritis (OA) has been rising exponentially in recent years. As the disease progresses, patients may eventually require surgical intervention to restore the functionality of the affected knees. The current literature review aims to explore two treatment options in regenerative medicine for OA by analyzing the efficacy and safety of platelet-rich plasma (PRP) and mesenchymal stem cells (MSCs) use, as well as determining which population will benefit from these treatments. A total of 1093 patients who were diagnosed with unilateral or bilateral knee osteoarthritis (KOA) were recruited in 23 studies. The experimental groups received either PRP or MSCs injections in comparison to the control groups receiving either hyaluronic acid (HA) or placebo (saline or dextrose) injections. Western Ontario and McMaster Universities Arthritis Index (WOMAC) was used to evaluate all participants at different time intervals of the studies. Medical imaging evaluations (X-ray or MRI) were used to look for structural improvements. In conclusion, both PRP and MSCs treatments were well tolerated, effective and safe to use. Repeated administrations and higher concentrations resulted in superior clinical improvements. A decrease in cartilage loss was observed in some MSCs trials. No severe adverse effects were documented. PRP treatment proved to be more efficacious among patients with KOA Kellgren-Lawrence (KL) grade I-II, while MSCs treatment proved to be more beneficial among the KOA KL grade II-III group.

## Introduction and background

Osteoarthritis is an irreversible progressive degeneration of a synovial joint (Table 1) that greatly affects the daily performance and the quality of life in the affected individuals. Knee osteoarthritis (KOA) is by far the most common type of arthritis diagnosed, with a rising prevalence due to an increase in the average lifespan and the obesity rate in the general population [1]. National statistics analyzed by the Centers for Disease Control and Prevention (CDC) reported that the prevalence of arthritis in adults aged 18 and older in the United States between 2013 and 2015 was estimated at around 54.4 million, which affected 22.7% of the general population [2]. Among the geriatric population aged 70 and above, the prevalence of KOA rises to 40% [3]. The corresponding projected prevalence may go up to 62.7 million by 2020 and 78.4 million by 2040 [4].

**Table 1:** Histopathological changes in knee joints among the aging population and osteoarthritis (OA) patients [1]

Cartilage changes	In aging population (aged 65 and above)	In OA patients
Chondrocyte size	Increased	Same
Chondrocyte count	Decreased	Same
Collagen	Same	Disorganized
Modulus of elasticity	Increased	Decreased
Proteoglycan content	Decreased	Decreased
Proteoglycan synthesis	Same	Increased
Water content	Decreased	Increased

Management of KOA depends on the severity of the disease and aims to reduce pain and improve physical functionality of the affected knees. Lifestyle modifications and conservative treatments like non-steroidal anti-inflammatory drugs (NSAIDs) and intra-articular injections using corticosteroid (CS) or hyaluronic acid (HA) are commonly used in managing mild KOA. However, these oral medications and intra-articular injections provide temporary pain relief only and often require frequent administrations for symptomatic control. They are not beneficial in severe cases. Neither CS nor HA therapy can reverse the preexisting damage in the affected synovial joints. Their corresponding potential adverse effects include muscle atrophy, cartilage damage, and toxicity [5], which may cause more harm than benefit for the patient. Ultimately, patients may require invasive procedures such as total knee arthroplasty (TKA). Unfortunately, not all patients are eligible candidates for surgeries, like patients who cannot tolerate general anesthesia or prolonged surgical procedures or with certain underlying medical conditions. 

The rise of regenerative medicine may provide promising treatment options for KOA patients. By far, the most promising alternatives are platelet-rich plasma (PRP) and mesenchymal stem cells (MSCs) injections, in terms of pain alleviation, restoration of the functional capacity [6], as well as potential tissue repairment [7]. In this literature review, the efficacy and safety of PRP and MSCs injections in KOA will be evaluated. We also aim to explore which population group will benefit from these treatment options.

## Review

Search strategy

This literature retrieval was performed by using the PubMed database search to obtain relevant articles dated up to September 1, 2020, that met the predefined inclusion criteria. The following keywords were used: knee osteoarthritis, intra-articular injection, platelet-rich plasma, and stem cell. Keywords searched under all fields yielded a wider selection of articles; however, applying Medical Subject Heading (MeSH) terms in search of literature retrieval resulted in a more comprehensive yet precise selection of relevant articles over a specific topic. For instance, the MeSH term “stem cell” was applied and all the related topics such as “progenitor cell”, “mother cell”, etc. were included in the search result. Therefore, MeSH keywords were adopted in articles selection for data collection (Table 2).

**Table 2:** Search results from PubMed database using regular keywords versus Medical Subject Heading (MeSH) keywords

Keywords used	Number of results searched by regular keywords	Number of results searched by MeSH keywords
Knee osteoarthritis, Intra articular injection, Platelet rich plasma.	237	146
Knee osteoarthritis, Intra articular injection, Stem cell.	207	38

Inclusion Criteria

Text Availability: Free full-text articles; Language: English literature; Subjects: Human beings

Exclusion Criteria

Language: Non-English literature; Subjects: Animals; Duplicated articles

Search result

**Figure 1:**
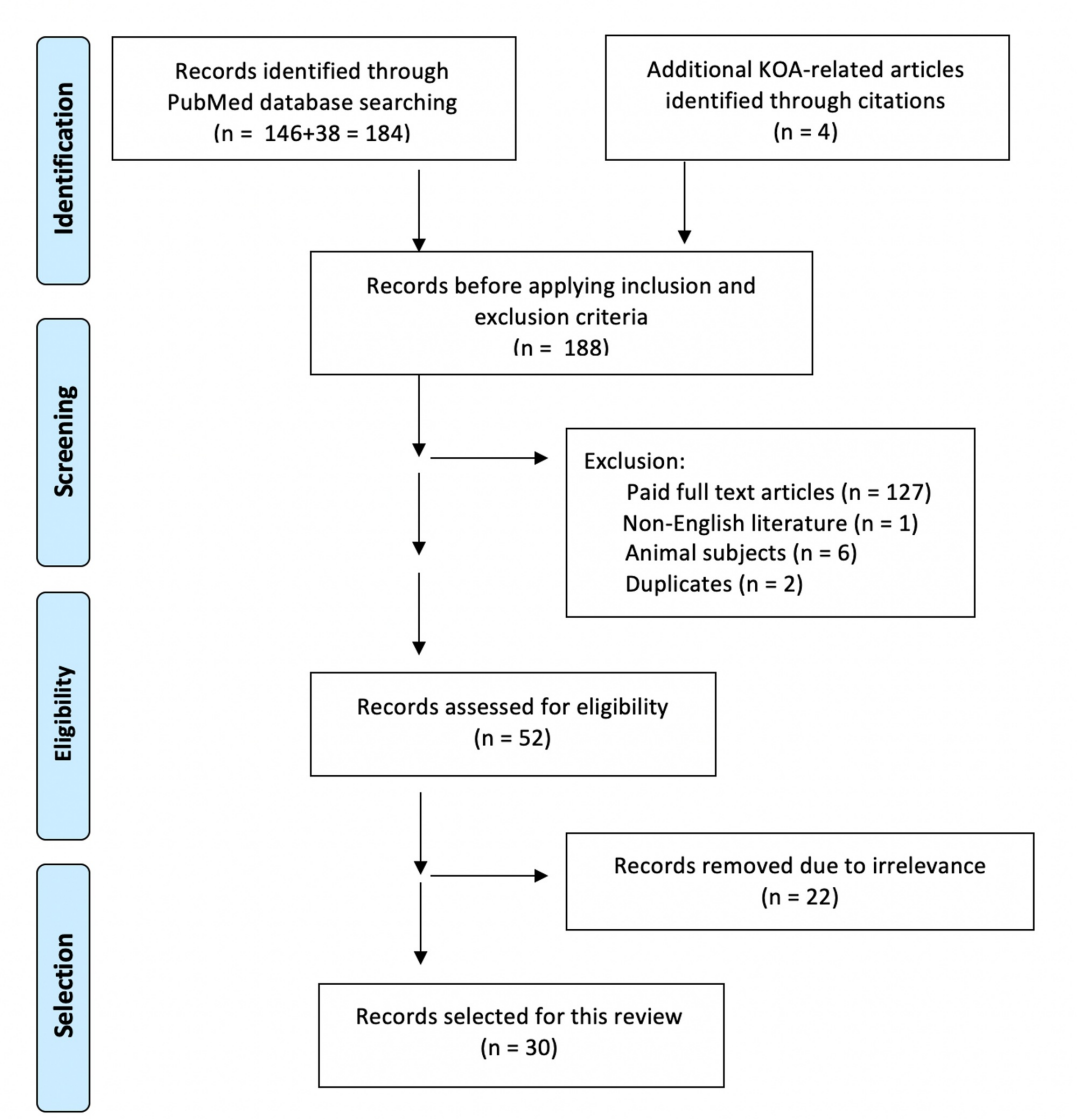
Preferred Reporting Items for Systematic Reviews and Meta-Analyses (PRISMA) flow diagram of topic search results and article selection process KOA: knee osteoarthritis

A collection of 146 articles related to platelet-rich plasma and 38 articles related to stem cell resulted in a total of 184 articles identified. Four additional articles of relevance were manually selected by reviewing the references of the selected articles. Two articles were removed due to duplication. After applying the predefined inclusion and exclusion criteria, 52 articles were assessed for eligibility. The eligibility of studies was determined by the authors after reviewing the full text of each article independently in our validation process. Twenty-two articles of irrelevance were removed. Therefore, a total of 30 articles were selected for this literature review (Table 3, Figure 1). 

**Table 3:** Screening process of article selection by applying inclusion and exclusion criteria

	Number of articles related to Platelet rich plasma	Number of articles related to Stem cell
Number of Records identified	146	38
Limited to free full text	35	22
Limited to English language	34	22
Limited to human subjects	32	18

Analysis 

In this literature review, only primary data were extracted from the original articles and analyzed as the sources of data were clearly stated; thus, a high reliability of the data is provided (Table 4). Systematic reviews and meta-analysis literature were considered secondary data analysis and therefore were not used in this review. 

**Table 4:** Summary of 23 clinical studies analyzed in this literature review CT: Clinical Trials, RCT: Randomized Controlled Trials, PRP: Platelet Rich Plasma, MSCs: Mesenchymal stem cells, HA: Hyaluronic Acid, NSAIDs: non-steroidal anti-inflammatory drugs, WOMAC: Western Ontario and McMaster Universities Arthritis Index

			Experimental group		Control group		Clinical Outcome assessed by WOMAC osteoarthritis index		
Studies	Type of Study Design	Number of participants (n=)	PRP	MSCs	HA	Placebo (Saline/Dextrose/NSAIDs)	PRP/MSCs are superior	No significant difference	PRP/MScs are inferior
(Rahimzadeh P, 2018) [8]	CT	42	✓			✓	✓		
(Taniguchi Y, 2018) [9]	CT	10	✓				✓		
(Sánchez M, 2019) [10]	CT	60	✓				✓		
(Sánchez M, 2016) [11]	CT	14	✓				✓		
(Buendía-López D, 2018) [12]	RCT	106	✓		✓		✓		
(Guillibert C, 2019) [6]	CT	57	✓				✓		
(Simental-Mendía M, 2019) [13]	RCT	35	✓				✓		
(Rasheed N, 2019) [14]	CT	214	✓				✓		
(Huang PH, 2017) [15]	CT	127	✓				✓		
(Sucuoğlu H, 2019) [16]	CT	42	✓				✓		
(Filardo G, 2012) [17]	RCT	109	✓		✓		✓		
(Lee WS, 2019) [18]	RCT	12		✓		✓	✓		
(Pers YM, et al., 2016) [19]	CT	18		✓			✓		
(Lamo-Espinosa JM, 2018) [20]	RCT	30		✓	✓		✓		
(Freitag J, 2019) [21]	RCT	30		✓		✓	✓		
(Al-Najar M, 2017) [22]	CT	13		✓			✓		
(Matas J, 2019) [23]	RCT	29		✓	✓		✓		
(Kuah D, 2018) [24]	RCT	20		✓		✓	✓		
(Lu L, 2019) [7]	CT	53		✓	✓		✓		
(Jo CH, 2014) [25]	CT	18		✓			✓		
(Chahal J, 2019) [26]	CT	12		✓			✓		
(Delgado-Enciso I, 2018) [27]	CT	24		✓		✓	✓		
(Pers YM, 2018) [28]	CT	18		✓			✓		
Subtotal	23 trials	1093							

The effectiveness of symptomatic control in pain relief and stiffness alleviation can be reflected by the level of improvement in physical function in patients suffering from KOA. Western Ontario and McMaster Universities Osteoarthritis Index (WOMAC score) [29] was the most commonly used assessment tool in evaluating the level of pain perception in patients with KOA among all the trials studied in this review. The questionnaire is divided into three subscales and scored by the sum of each subscale: 0-20 for Pain, 0-8 for Stiffness, and 0-68 for Physical Function.

Plain X-ray and magnetic resonance imaging (MRI) were used in evaluating the disease progress by measuring the thickness of cartilage in tibial and femoral subregions at baseline and after treatments. 

Discussion

In the analysis of 1093 patients among 23 trials, the efficacy and safety of intra-articular injections of platelet-rich plasma (PRP) and mesenchymal stem cells (MSCs) were thoroughly evaluated and studied. Based on the severity of articular damage and the limitation of physical functionality in the recruited patients, they were categorized into mild KOA group with Kellgren-Lawrence (KL) grade I-II or moderate to severe KOA group with KL grade III-IV. Both PRP and MSCs treatments were beneficial to patients diagnosed with mild to moderate KOA. Beneficial clinical outcomes were observed by means of lowered pain perception and increased physical functionality; therefore, the quality of life in KOA patients had been significantly improved. 

This review aimed at analyzing the efficacy and safety of PRP and MSCs treatments, as well as determining the indications of these treatments. A total of 23 studies were analyzed in this review, which were summarized in Table 4 above. 

Characteristics of participants in the included studies: female to male ratio was higher, up to 3:1 [15]. Possible reasons would be the dropout rate of male patients tended to be higher, the prevalence of KOA could be higher among the female population due to genetic composition, female patients were more likely to seek medical attention at an earlier stage of the disease and were willing to participate in clinical trials or studies. 

Hyaluronic acid (HA)

HA injections has been widely used for pain relief in KOA patients for the past three decades [30]. In five studies, HA was used as the control group in comparison to the experiment group of PRP or MSCs [7,12,17,20,23]. 

Mechanism of Action

HA injections aim to increase the total intra-articular volume in the synovial joint which reduces the intensity of friction between articular surfaces. HA acts as a lubricant which increases the viscosity and elasticity in the synovial fluid; thereby, reduces the pain and further cartilage damage caused by KOA [31]. The major concern of HA treatment is the wear off effect over time, which requires frequent administration for temporary pain relief [12,17]. Therefore, the search for regenerative medicine is the mainstream of alternative treatment for KOA patients. 

Platelet-rich plasma (PRP)/autologous conditioned plasma (ACP)

PRP, also referred to as ACP, as the name suggests, is derived from oneself and is prepared for therapeutic purpose. The efficacy of PRP has been studied over the past two decades [9,14]. In this review, there was a general consensus among the studies that PRP treatments proved to be effective for KOA.

Mechanism of Action

PRP possesses anti-inflammatory properties that can reduce the level of systemic inflammation in KOA by inhibiting the release of matrix metalloproteinase (MMP-9) and cytokines such as tumor necrosis factor (TNF-α) and interleukins (IL-1β) [12]. 

Preparation of PRP

There is no universal guideline in the treatment source or preparation methods, which makes it hard to compare the efficacy among different trials or studies (Table 5). In general, fresh venous blood samples were collected via venipuncture, which underwent the process of centrifugation in order to obtain PRP in the included studies. Three studies adopted a double centrifugation method to eliminate erythrocytes in the first round and concentrate platelets in the sample in the second round [12,14,18]. After centrifugation twice, the PRP samples were more purified and beneficial from a lowered risk of inflammation induced by leukocytes. The speed and timing for the centrifugation process were the determinants of the variations which might affect the end result of the sample; therefore, a universal standardized protocol was in need to create homogeneity in treatment.

**Table 5:** Summary of platelet-rich plasma (PRP) preparation methods used in clinical trials

Trials	Source of PRP	Volume of blood collected (mL)	Number of spins of centrifugation	Volume of PRP used for injection (mL)
(Buendía-López D, 2018) [12]	Antecubital vein	60	2	5
(Guillibert C, 2019) [6]	Vein	18	1	8.8
(Simental-Mendía M, 2019) [14]	Vein	45	2	5
(Filardo G, 2012) [18]	Vein	150	2	5, 5, 5

Three studies selected leukocyte-poor PRP injections as the experimental group based on a research finding that leukocyte-poor PRP showed reduced inflammatory features (tenderness and swelling) than leukocyte-rich PRP [12,16,17]. This resulted in superior clinical enhancement compared to the control group using HA injections or NSAIDs therapy. Gentler immune responses resulted and less discomfort was experienced, which made leukocyte-poor PRP a more favorable treatment option for KOA patients.

One study used the freezing method to preserve PRP for quality control and homogeneity in consecutive treatments [18]; however, the biological activity of platelets undergoing the freezing and thawing processes could be compromised due to degranulation. This can be a major breakthrough in the production of PRP. Standardized measures are needed to ensure the homogeneity and quality control of PRP products in the future. Massive production can lower the total cost of production, which may result in a reduced treatment cost and make it more affordable for the affected population. Nevertheless, further studies are required to prove the efficacy of frozen PRP compared to fresh PRP. 

Route of Administration

In all clinical trials listed in Table 4, PRP was administrated through intra-articular injections; however, one study suggested that a combination of intra-articular (IA) and intra-osseous (IO) administration of PRP in patients with severe KOA of KL grade III-IV had provided a superior efficacy in long-term symptomatic relief as well as significant improvements in physical functionality at six- and 12-month intervals compared to the IA group [10]. However, no significant difference was observed at the two-month interval between the IA and IO groups in short-term pain control. No additional adverse effects were recorded in the IO group. Disadvantages of IO administration included the requirement of procedural sedation and anesthesia in the operation room, which undermined the accessibility of treatment in an outpatient setting; secondly, the total cost of treatment would increase due to the need for the prerequisite procedure, which made this treatment option less economical. In addition, IO administration led to extensive post-procedural discomfort and resulted in a longer recovery time when compared to IA injections. The cost-effectiveness of the IO administration should be further evaluated in future studies. 

Clinical Efficacy 

One study concluded that PRP treatment is more effective in pain reduction at six months and 12 months than HA use (p=0.02) [12]. One study showed that using a large volume (8 mL) of pure PRP compared to a small volume (3-5.5 mL) in one single injection generated a significant clinical improvement with a response rate of 80% up to six months post-injection [6]. Two studies indicated that triple administrations of PRP at monthly intervals yielded a more satisfactory effect in pain control based on visual analog scale (VAS) and WOMAC pain scores then single application (p=0.0007) [14,16]. Two studies concluded that the PRP treatment was not beneficial in patients with moderate to severe KOA, which was classified as Kellgren-Lawrence grade III-IV, as there was no advantage over the HA treatment in that particular population [17,18]. The effectiveness of PRP treatment in KOA patients was undoubtedly positive, strongly supported by all the listed clinical trials in Table 4. It worked best among patients diagnosed with KOA Kellgren-Lawrence Grade I-II, probably due to its anti-inflammatory characteristics. Increased dosage of PRP by repetitive administrations or larger PRP volume yielded a better clinical outcome, which suggested that the effectiveness of PRP might be dose-dependent; however, the optimal therapeutic dosage has yet to be established in future studies. Although there was no conclusive evidence that PRP had regenerative properties but unlike steroids it was not associated with cartilage damage. However, in severe KOA patients of Kellgren-Lawrence Grade III-IV, PRP treatment showed minimal clinical improvements; thereby, it was not recommended for that particular population.

One study concluded that neither improvement nor deterioration was seen on medical imaging from baseline to week 52 in the experimental group. No changes in cartilage thickness observed; thus, no reduction of disease progression based on Kellgren-Lawrence classification [12]. The observation period in this study was relatively short, and the full picture of the clinical efficacy might not be obtained in one year’s time. For instance, the effect of PRP might not have reached the peak by the end of week 52. The regenerative potential of PRP remains doubtful and more studies are needed in the future to completely explore its regenerative properties. 

Safety

The most common adverse effect of PRP use was mild local tenderness after injections which spontaneously resolved within 24-48 hours without intervention [9]. One study compared the post-injective pain reactions between the PRP group and the HA group in terms of level of tenderness and swelling. The data showed that the PRP group had a significantly higher level of immediate pain while both groups shared similar swelling features after treatments (p=0.039) [21]. The possible cause could be due to the presence of erythrocytes or leukocytes residue in PRP, causing local irritation and possible inflammation. 

Mesenchymal stem cells (MSCs)

The feasibility of MSCs use as an advanced treatment option of regenerative medicine in treating KOA and other structural damage has been studied in multiple trials over the past decade [21,25]. There were numerous studies conducted on animals or performed in vitro; however, limited number of clinical trials on humans were carried out and the sample size was mostly small [18,19,22,25,26,28]. 

Mechanism of Action

The effect of MSC treatment implies an anti-inflammatory property by inhibiting the maturation of immune cells: lymphocytes and monocytes. As a result, the suppression of natural killer cells, dendritic cells, macrophages and cytotoxic T cell prevents the activation of unwanted immune response and cell apoptosis in the affected joint, which theoretically prevents cartilage damage from disease progression. Endogenous stem cells are being investigated for their regenerative properties in tissues: proliferation and differentiation into collagen and extracellular matrix in the process of chondrification [24]. The mechanism of action of MSCs has not been sufficiently studied and further investigations are needed in the future studies.

Source and Preparation for Stem Cell Harvesting

Adipose tissue was commonly used as the source of MSCs due to their abundancy and accessibility in the human body. Bone marrow and umbilical cord were used in other studies to explore and compare the efficacy of stem cells from different origins (Table 6).

**Table 6:** Source and Preparation for Stem Cell harvesting N/A stands for Not Available. MSCs: mesenchymal stem cells

Trials	Source of MSCs	Amount of sample collected	Days required for cell preparation	Dosage of MSCs used for injection (×10^6^ cells)
(Pers YM, et al., 2016) [20]	Adipose tissue	10 grams	14	2, 10, 50
(Lamo-Espinosa JM, 2018) [21]	Bone marrow	N/A	N/A	10, 100
(Freitag J, 2019) [22]	Adipose tissue	60 mL	N/A	N/A
(Al-Najar M, 2017) [23]	Bone marrow (from iliac crest)	35-40 mL	N/A	30
(Matas J, 2019) [24]	Umbilical cord	N/A	N/A	20
(Kuah D, 2018) [25]	Adipose tissue	N/A	N/A	3.9, 6.7 (in 2mL)
(Lu L, 2019) [26]	Adipose tissue	N/A	N/A	50

In four studies, autologous adipose tissues were collected by the liposuction procedure and underwent collagenase digestion in order to obtain stromal vascular fraction (SVF), a cellular component of the lipoaspirate [20,22,25,26]. Stem cells were harvested and evaluated by microbiological analysis and reverse transcription polymerase chain reactions (rt-PCR). After 14 days of culture, homogenization process was performed for quality control in terms of cell phenotyping, viability, and count [30]. Sterility was achieved by testing negative for toxin and bacterial contaminations [23].

One study used the MSCs from one single donor to achieve quality control and homogeneity of the experimental treatment. The harvested MSCs were preserved by deep freezing at -150°C and were thawed prior to usage [28]. 

Clinical Efficacy 

One study resulted in significant clinical improvement among the experimental group using low-dose adipose-derived (AD) MSCs treatment (2×10^6^ cells) one week after intra-articular injection (p<0.001), which was evaluated by WOMAC score. Clinical improvement was observed among the high-dose MSCs treatment group (50×10^6^cells) three weeks after injection; however, the results in the high-dose groups were not statistically significant (p=0.11 and p=0.99) [25]. Contrarily, another study suggested that using higher-dose MSCs (100×10^6^ cells) yielded a better statistical outcome (p=0.004) [21].

One study suggested that repeated dosage of MSCs use yielded a better clinical outcome in pain alleviation by 86% and functionality improvement by 89% when compared to the control group using hyaluronic acid (HA) injections (p=0.001) [23]. The effect of HA injections lasted for six months only. In comparison, the effect of a single administration of MSCs treatment lasted for nine months on average, while the repeated administration of MSCs treatment extended the effect over 12 months with steady improvements observed. The use of analgesics could be a confounding factor in pain reduction among the experimental and control groups. The proliferative and differentiative properties of MSCs are yet to be established and proven histologically by further studies. 

Four studies indicated that there was an increased synovial volume in affected knee joints observed after MSCs treatments [7,11,25,28]. A significant increase in bilateral femoral cartilage resulted (p=0.0086 on left knees, p=0.0038 on right knees) at 12-month intervals after MSCs treatment [28]. However, one study concluded that no further damage, calcification, or structural improvements was observed in MRI studies [23]. One study indicated that the administrations of low-dose MSCs (3.9×10^6 ^cells) yielded a better result in the reduction of cartilage loss over the lateral tibial area than high-dose MSCs (6.7×10^6^ cells) group [25].

Safety 

Bruising and local discomfort were commonly noted as the related side effects after liposuction procedures and injections. They were self-limiting and resolved spontaneously over a short period of time (within 48 hours). Cold compression and oral analgesics were sometimes used for symptomatic relief. Arthralgia was the most common reported adverse effect in the experimental group versus the placebo group [26]. However, two cases of patellar bursitis were recorded in one study, which eventually resolved in two weeks [27]. The possibility of microbial infection was ruled out as the culture results came back negative. The etiology could be procedural-related or local allergic reactions; therefore, further investigations were needed to evaluate the safety of MSCs application. 

One study revealed that half of the experimental group of single injection of AD-MSCs developed osteophyte progression at 12 months; meanwhile, 89% of the double injection group did not have osteophyte progression, which aimed for better KOA stabilization. These results were supported by MRI analysis. In addition, the double injection group experienced an increase in moderate adverse effects at the six-month interval [24]. 

Limitations

The major limitation of this review was the accessibility of paid literature articles. Only free full-text articles were included as a result of economical hinderance. Non-English literature was removed to avoid translational error or misinterpretation, which reduced the total sample size of all clinical studies; therefore, it underestimated the actual clinical data. Animal studies were not included due to the lack of subjective feedback and the existence of biological differences in different animal species from the human body. Yet, numerous MSCs studies were carried out in animals such as mice and rabbits. In most MSCs clinical trials, the small sample size and the lack of control group undermined the statistical power of the study. It is unethical to perform invasive procedures such as liposuction or bone marrow aspiration, only for the patient to find out that they were in the control group receiving a placebo that is not beneficial or favorable to their underlying condition; thus, this could be the main cause of the absence of a formal placebo for MSCs. Most of the studies were not double-blinded; therefore, the skills and judgement power of the physician might be biased.

## Conclusions

In the analysis of 30 published articles on PubMed, this study concludes that both intra-articular platelet-rich plasma (PRP) and mesenchymal stem cells (MSCs) injections are effective in patients with mild to moderate KOA in terms of providing symptomatic relief, restoring physical functionality and improving potential tissue repairment in the affected joints. PRP treatment works best among patients with KOA KL grade I-II, while MSCs treatment works best among the KOA KL grade II-III group. Both treatments show minimal effects in patients with severe KOA of KL grade IV. Repetitive administrations and the application of larger test volume result in a more favorable clinical outcome in both treatments. They are well tolerated without serious adverse effects observed. Regenerative medicine has shown promising superior results in both therapeutic effectiveness and safety when compared to traditional conservative treatments such as hyaluronic acid injections. However, the mechanism of actions of PRP and MSCs remains unclear and further studies are needed to establish a standardized preparation method, to determine the optimal dosage and the frequency of usage. Large-scale randomized double-blind controlled trials are recommended in the future.
